# Topoisomerase II alpha gene amplification is a favorable prognostic factor in patients with HER2-positive metastatic breast cancer treated with trastuzumab

**DOI:** 10.1186/1479-5876-10-212

**Published:** 2012-10-23

**Authors:** George Fountzilas, Christos Christodoulou, Mattheos Bobos, Vassiliki Kotoula, Anastasia G Eleftheraki, Ioannis Xanthakis, Anna Batistatou, George Pentheroudakis, Nikolaos Xiros, Irene Papaspirou, Anna Koumarianou, Pavlos Papakostas, Dimitrios Bafaloukos, Dimosthenis V Skarlos, Konstantine T Kalogeras

**Affiliations:** 1 Department of Medical Oncology, “Papageorgiou” Hospital, Aristotle University of Thessaloniki School of Medicine, 564 03, Thessaloniki, Macedonia, Greece; 2 Second Department of Medical Oncology, “Metropolitan” Hospital, Athens, Greece; 3 Laboratory of Molecular Oncology, Hellenic Foundation for Cancer Research, Aristotle University of Thessaloniki School of Medicine, Thessaloniki, Greece; 4 Department of Pathology, Aristotle University of Thessaloniki School of Medicine, Thessaloniki, Greece; 5 Section of Biostatistics, Hellenic Cooperative Oncology Group, Data Office, Athens, Greece; 6 Department of Pathology, Ioannina University Hospital, Ioannina, Greece; 7 Department of Medical Oncology, Ioannina University Hospital, Ioannina, Greece; 8 Oncology Section, Second Propaedeutic Department of Internal Medicine, University General Hospital “Attikon”, Athens, Greece; 9 Histopathology Department, “Alexandra” Hospital, Athens, Greece; 10 Department of Medical Oncology, “Hippokration” Hospital, Athens, Greece; 11 First Department of Medical Oncology, “Metropolitan” Hospital, Athens, Greece; 12 Translational Research Section, Hellenic Cooperative Oncology Group, Data Office, Athens, Greece

**Keywords:** Breast cancer, Topoisomerase II alpha, Fluorescence in situ hybridization, Gene amplification, Trastuzumab, Prognostic factors, Anthracyclines, Predictive factors

## Abstract

**Background:**

The vast majority of patients with HER2-positive metastatic breast cancer (MBC) treated with trastuzumab eventually develop resistance to this agent. There is an unmet need therefore, for identifying biological markers with possible prognostic/predictive value in such patients. The aim of this study was to investigate the prognostic role of topoisomerase II alpha gene (*TOP2A*) amplification and protein (TopoIIa) expression in patients treated with trastuzumab-containing regimens.

**Methods:**

Formalin-fixed paraffin-embedded tumor tissue samples were retrospectively collected from 225 eligible patients treated with trastuzumab. Protein expression of ER, PgR, Ki67, PTEN, HER2 and TopoIIa were centrally assessed by immunohistochemistry. *HER2* and *TOP2A* gene amplification was evaluated by fluorescence in situ hybridization. *PIK3CA* mutations were identified by single nucleotide polymorphism genotyping. Survival was evaluated from the initiation of trastuzumab as 1st line treatment to the date of last follow-up or death.

**Results:**

Among the 225 samples analyzed, only 137 (61%) were found to be HER2-positive. *TOP2A* was amplified in 41% and deleted in 16% of such tumors. *TOP2A* gene amplification was more frequent in ER-negative tumors. TopoIIa protein expression was observed in the majority (65%) of the samples and was associated with ER-positive status, high Ki67 expression, presence of PTEN protein and *PIK3CA* mutations. Median follow-up for patients treated in the 1st line was 51 months. Survival was more prolonged with trastuzumab-containing treatment in HER2-positive patients (50 months, log-rank, p=0.007). *TOP2A* non-amplified or deleted tumors were associated with increased risk for death compared to *TOP2A* amplified tumors (HR=2.16, Wald’s p=0.010 and HR=2.67, p=0.009, respectively). In multivariate analysis, a significant interaction of *TOP2A* with anthracycline treatment (either in the adjuvant or the 1st line setting) was observed for survival (Wald’s p=0.015). Among the *TOP2A* amplified subgroup, anthracycline-treated patients were associated with decreased risk for death.

**Conclusions:**

*TOP2A* gene amplification was shown to be a favorable prognostic marker in HER2-positive MBC patients treated with trastuzumab, such an effect however, appears to rather be related to treatment with anthracyclines (predictive marker for benefit from anthracyclines). The results of the present retrospective study warrant validation in larger cohorts of patients treated in the context of randomized trials.

## Background

Metastatic breast cancer (MBC) is an incurable disease. Chemotherapy or hormonal therapy mainly has a palliative role, although newer agents may contribute to a significant prolongation of survival [1,2]. *HER2*, a proto-oncogene located on chromosome 17q21.1, is amplified in approximately 20% of breast cancers and is associated with a number of adverse prognostic factors, such as axillary node involvement, advanced stage, hormone receptor (HR)-negativity and increased proliferation indices [3,4]. Trastuzumab (Herceptin®, Genentech, San Francisco, CA), a recombinant humanized monoclonal antibody against the HER2 protein, was found to prolong progression-free survival (PFS) and overall survival (OS) of patients with MBC and *HER2* gene amplification or HER2 protein overexpression [5,6].

Nevertheless, it has become evident from numerous clinical trials that a considerable number of patients with MBC do not benefit from the administration of trastuzumab, either as a single agent or in combination with other systemic treatments. Moreover, in almost all patients who initially respond to trastuzumab-based treatments, tumor progression is eventually expected to occur. On the other hand, it is conceivable that any given targeted treatment is cost-effective only when it is administered exclusively to those patients who will derive the greatest benefit from it, sparing all other patients from unnecessary side effects. Therefore, there is an imperative need for identifying biological markers that will predict which patients are most likely to respond to trastuzumab-based treatments.

The topoisomerase II alpha gene (*TOP2A*) is located telomerically to *HER2* at 17q21-q22 and encodes for topoisomerase II alpha (TopoIIa), a 170-kd cell cycle regulated protein [7]. The TOP2A gene is considered to be within the HER2 amplicon [8], although it is not included in the “smallest region of amplification” [9] and may follow a different fate than HER2 in terms of copy number alterations [10]. Topoisomerases II are considered to be targets of anthracyclines [11], while *TOP2A* gene amplification has been linked to anthracycline sensitivity in patients with advanced breast cancer [12,13] or in patients with high-risk primary breast cancer receiving adjuvant chemotherapy [14-16] (reviewed in [17]). On the other hand, the predictive value of *TOP2A* has been refuted by several retrospective studies [18-20] however, flaws in the statistical design and the methodological approaches used in these studies undermine the credibility of such opinions. In fact, a number of investigators believe that TopoIIa protein expression is more relevant than *TOP2A* gene status in predicting response to anthracyclines [21].

It is noteworthy, that although extensive research efforts have been devoted to the evaluation of the potential predictive role of *TOP2A* gene status with respect to anthracycline responsiveness, little attention has been paid to *TOP2A* gene alterations with regard to the outcome of breast cancer patients following treatment with trastuzumab in advanced stages. The main objectives of the present study were to explore the impact of TOP2A gene status and TopoIIa protein expression on the outcome of MBC patients treated with trastuzumab-containing regimens and their possible interaction with anthracycline-containing treatment. The study was retrospective in nature and was performed on archival tissue material (formalin-fixed paraffin-embedded, FFPE) from our Group’s Tumor Repository. In addition, based on the adverse prognostic effect of PIK3CA mutations and PTEN protein loss in the same patient cohort, as previously shown [22], we investigated the association of these parameters with TOP2A gene and protein status.

## Patients and methods

The medical records of all patients with MBC treated with trastuzumab-based regimens, between December 1998 and January 2010, were retrospectively reviewed. Eligibility criteria for this study were a: histologically confirmed MBC; b: adequacy of clinical data on patient’s history, demographics, tumor characteristics, treatment details (drug dosages, schedule of administration, serious toxicities) and clinical outcome; c: availability of adequate FFPE tumor tissue for biological marker evaluation; and d: trastuzumab-based treatment for metastatic disease. The translational research protocol was approved by the Bioethics Committee of the Aristotle University of Thessaloniki School of Medicine (Protocol # 4283; Jan 14, 2008) under the general title “Investigation of major mechanisms of resistance to treatment with trastuzumab in patients with metastatic breast cancer”. All patients included in the study after 2005 provided written informed consent for the provision of biological material for future research studies before receiving any treatment. Waiver of consent was obtained from the Bioethics Committee for patients included in the study before 2005.

### Tissue Material

**Figure 1:**
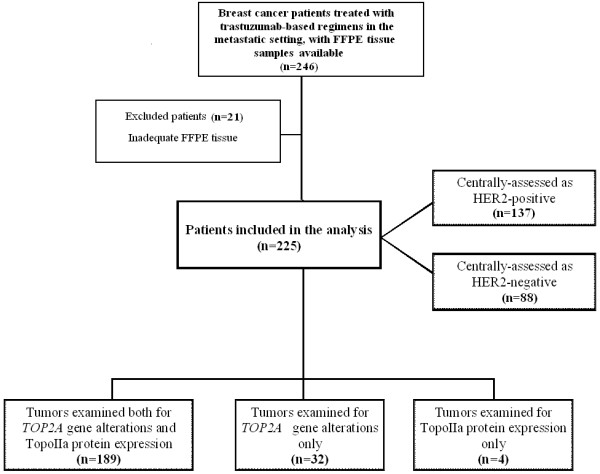
**REMARK diagram.** FFPE tissue availability in the present study for the application of different analytical techniques for the determination of *HER2* and *TOP2A* gene amplification status and HER2 and TopoIIa protein expression is presented in detail.

FFPE tumor tissue samples were retrospectively collected from 246 breast cancer patients treated with trastuzumab-based regimens in the metastatic setting. Twenty-one cases were excluded for inadequate FFPE tumor tissue, thus decreasing the number of eligible/evaluable patients to 225. A REMARK diagram for the translational research studies is provided in Figure 1. Representative hematoxylin-eosin stained sections from the tissue blocks were reviewed by a pathologist (M.B.). The most representative tumor areas were marked for the construction of tissue microarray (TMA) blocks, as previously described [22]. Each TMA block also contained cores from various neoplastic, non-neoplastic and reactive tissues serving as assay controls. Cases not represented, damaged or inadequate on the TMA sections were re-cut from the original blocks and these sections were used for protein and gene analysis. 

### Immunohistochemistry (IHC)

Immunohistochemical labeling was performed according to standard protocols on serial 2.5 μm thick sections from the original blocks or the TMA blocks. All cases were also stained for vimentin (clone V9, Dako, Glostrup, Denmark) and cytokeratin 8/18 (clone 5D3, Novocastra™, Leica Biosystems, Newcastle, U.K), which were used as control stains for tissue immunoreactivity and fixation, as well as identification of tumor cells. Tissue samples negative for the above antibodies were excluded from the study. The staining procedures for estrogen receptor (ER, clone 6F11, Novocastra™, Leica Biosystems), progesterone receptor (PgR, clone 1A6, Novocastra™, Leica Biosystems), HER2 (A0485 polyclonal antibody, Dako) and Ki67 (clone MIB-1, Dako) were performed using a Bond Max™ autostainer (Leica Microsystems, Wetzlar, Germany), as previously described [23]. TopoIIa protein expression was evaluated using the KiS1 monoclonal antibody (Dako), as previously described [24] with slight modifications (antibody dilution: 1:200; detection system: Envision™, Dako). PTEN (phosphatase and tensin homologue deleted on chromosome 10) protein expression was evaluated using the 6H2.1 monoclonal antibody (Dako), as previously described [25]. The evaluation of all IHC sections was done by experienced in breast cancer pathologists (M.B. and A.B.), blinded as to the patients’ clinical characteristics and survival data. To assure optimal immunoreactivity, the sections of the TMA blocks were stained in one run for each antibody, shortly after mounting of the TMA sections on positively charged glass slides.

### Interpretation of the IHC results

ER and PgR, HER2, Ki67 and PTEN immunostaining was evaluated according to existing established criteria [26-29], as previously described [22]. Briefly, ER and PgR were evaluated using the Histoscore method (max score: 400) and were considered positive if staining was present in ≥1% of tumor cell nuclei [26]; HER2 protein expression was scored in a scale from 0 to 3+, the latter corresponding to uniform, intense membrane staining in >30% invasive tumor cells [27]; for Ki67, the expression was defined as low (<14%) and high (≥14%) based on the percentage of stained/unstained nuclei from the tumor areas [28]; and, PTEN protein expression (cytoplasmic, nuclear or both) was evaluated according to a staining intensity scale from 0 (negative, no staining) to 3 (intense staining), whereby tumors with PTEN scores of 0 or 1 were considered to have PTEN loss [29]. For TopoIIa immunostaining, a tumor was considered to be positive if moderate to intense nuclear staining was detected in >5% of tumor cells [30].

### *Fluorescence* in situ *hybridization (FISH)*

TMA sections or whole sections (5 μm thick) were cut for FISH analysis, using the ZytoLight® SPEC *HER2*/*TOP2A*/centromere 17 (CEN17) triple color probe kit (ZytoVision, Bremerhaven, Germany). FISH was performed according to the manufacturer’s protocol with minor modifications. Four carcinoma cell lines (MDA-MB-231, MDA-MB-175, MDA-MB-453 and SK-BR-3) from the Oracle HER2 Control Slide (Leica Biosystems), with a known *HER2* gene status, were also used as a control of the FISH assays and analyzed for *HER2* and *TOP2A* genomic status.

### FISH evaluation

For all probes, sequential (5 planes at 1.0 μm) digital images were captured using the Plan Apo VC 100x/1.40 oil objective (Nikon, Japan) using specific filters for each probe. The resulting images were reconstructed using specifically developed software for cytogenetics (XCyto-Gen, ALPHELYS, Plaisir, France). For the evaluation of *HER2*/*TOP2A*/CEN17 status, non-overlapping nuclei from the invasive part of the tumor were randomly selected, according to morphological criteria using DAPI staining, and scored (M.B.). The virtual slides of HER2, ER or PgR stains, created as previously described [31], were used for selecting the invasive part of the tumor in each TMA. Twenty tumor nuclei were counted according to Press et al. [13]. The *HER2* gene was considered to be amplified when the ratio of the respective gene probe/centromere probe was ≥2.2 [27] and deleted when the ratio was <0.75. The *TOP2A* gene was considered to be amplified when the ratio of the respective gene probe/CEN17 probe was ≥2.0 and deleted when the ratio was <0.8 [32]. In cases with values at or near the cut-off (1.8-2.2 for amplifications and 0.7-0.9 for deletions), additional 20 or 40 nuclei were counted and the ratio was recalculated. In cases with a borderline ratio at 60 nuclei, additional FISH assays were performed in whole sections. All primary image data of the TMA and whole tumor sections have been digitally scanned and made publicly available at: http://www.hecog-images.gr/readDir.php?nextdir=TOP2AtrastuzumabMBC.

### Single nucleotide polymorphism (SNP) genotyping for PIK3CA mutations

DNA was extracted from 182 FFPE whole tissue sections or macrodissected tissue fragments containing >70% tumor cells, using a fully automated isolation method based on silica-coated magnetic beads (Versant Tissue Preparation Reagents, Siemens Healthcare Diagnostics, Tarrytown, NY) in combination with a liquid handling robot, as previously described [33]. Mutation testing for *PIK3CA* E542K and E545K (exon 9) and H1047R (exon 20) was accomplished with custom Taqman-MGB-SNP genotyping assays (duplex q-PCR for the detection of control DNA and mutant target in the same reaction), as previously described [22].

### Statistical analysis

Data on selected patient and tumor characteristics, previous and subsequent lines of treatment, disease progression, events and survival were obtained from medical records and entered into a central database. Follow-up information was updated in February 2010. Associations between the examined markers were performed in the total cohort using the chi-square or Fisher’s exact tests where appropriate. The majority of patients received trastuzumab in the 1st line of treatment for metastatic disease and thus time to progression (TTP) was defined as the time from trastuzumab initiation in the 1st line of treatment (with or without concurrent chemo/hormonal therapy) to the date of documented disease progression. Survival was measured from the initiation of trastuzumab treatment in patients receiving trastuzumab as a 1st line treatment to the date of death. Patients alive were censored at the date of the last follow-up contact. Survival probabilities were estimated by the Kaplan-Meier method and compared using the log-rank test. For the univariate and multivariate analyses, Cox proportional hazards models were used. Univariate analyses were performed separately in HER2-positive and HER2-negative patients, while multivariate analyses were performed in the total cohort and in the population of clinical interest, i.e. the HER2-positive patients. Interaction tests for the examined markers (*TOP2A* gene status and TopoIIa protein expression) with HER2 and ER/PgR status were performed. We also examined the interaction of the examined markers with anthracycline-containing treatment in the adjuvant and/or the 1st line metastatic setting. In the multivariate setting, model choice was performed using backward selection criteria with p<0.10, including in the initial step clinico-pathological parameters, such as age (>60 vs. 50–60 vs. <50), menopausal status (post vs. pre), performance status (1–2 vs. 0), number of metastatic sites (≥3 vs. <3), anthracycline treatment (yes vs. no), hormonal receptor status (ER/PgR) (positive vs. negative), Ki67 protein expression (high vs. low), HER2 status (positive vs. negative), *TOP2A* gene status (deleted vs. non-amplified vs. amplified) and TopoIIa protein expression (positive vs. negative). Multivariate analyses were performed in the total cohort and in the HER2-positive subgroup and were presented by forest plots. All tests were two-sided at the α=0.05 level of significance. No adjustment for multiple comparisons was performed. Results of this study were presented according to reporting recommendations for tumor marker prognostic studies [34]. The SPSS (version 15.0, IBM Corporation, Armonk, NY) and SAS (version 9.3, SAS Institute Inc., Cary, NC) software were used for statistical analysis.

## Results

Among the 225 eligible patients with metastatic breast cancer treated with trastuzumab, only 137 (61%) were found to have centrally assessed *HER2* gene amplification by FISH and/or 3+ HER2 protein overexpression by IHC (Figure 1). It is of note that all 225 patients were considered to be HER2-positive when assessed with IHC (and FISH in some cases) at the local laboratories and had therefore been treated with trastuzumab. Selected patient and tumor characteristics from the 225 patients, at trastuzumab initiation, are presented in Table 1.

**Table 1:** Selected patient and tumor characteristics (at trastuzumab initiation) according to HER2 status

	**HER2 status**	
	**Positive**	**Negative**
**N**	137	88
Age (years)^1^		
Median (range)	54.6 (28.4-95.0)	58.9 (31.8-78.8)
	**N (%)**	**N (%)**
Menopausal status		
Premenopausal	43 (31.4)	26 (29.5)
Postmenopausal	94 (68.6)	62 (70.5)
Performance status		
0	96 (70.1)	56 (63.6)
1	28 (20.4)	18 (20.5)
2	5 (3.6)	7 (8.0)
Unknown	8 (5.8)	7 (7.8)
History of adjuvant CT	75 (54.7)	55 (62.5)
Anthracycline containing	59 (43.1)	29 (33.0)
Taxane containing	36 (26.3)	17 (19.3)
CMF-like	42 (30.7)	30 (34.1)
History of adjuvant HT	61 (44.5)	42 (47.7)
History of adjuvant RT	52 (38.0)	33 (37.5)
Tumor grade (initial diagnosis)		
1	4 (2.9)	2 (2.3)
2	48 (35.0)	36 (40.9)
3	75 (54.7)	42 (47.7)
Unknown	10 (7.3)	8 (9.1)
Site of metastases		
Locoregional	45 (32.8)	28 (31.8)
Distant	117 (85.4)	78 (88.6)
Only locoregional	10 (7.3)	5 (5.7)
Only distant	82 (59.9)	55 (62.5)
Bones	54 (39.4)	37 (42.0)
Visceral	93 (67.9)	58 (65.9)
Number of metastatic sites		
1	54 (39.4)	28 (31.8)
2	40 (29.2)	30 (34.1)
≥3	38 (27.7)	25 (28.4)
Unknown	5 (3.6)	5 (5.7)
History of 1st line CT	123 (89.8)	68 (77.3)
Anthracycline containing	18 (13.1)	9 (10.2)
Number of treatment lines with T		
1	51 (37.2)	39 (44.3)
2	33 (24.1)	19 (21.6)
3	23 (16.8)	12 (13.6)
≥4	30 (21.9)	18 (20.5)

^1^p=0.019; *CT* chemotherapy, *HT* hormonal therapy, *RT* radiotherapy, *T* trastuzumab.

HER2-positive:*HER2* amplification by FISH and/or HER2 3+ by IHC.

Trastuzumab was given as 1st line treatment in 191 patients (85%), while in 15% of the patients trastuzumab was initiated later in the course of metastatic disease. The majority of the 1st line treated patients received it in combination with chemotherapy (186 patients, 97%), while the rest (5 patients, 3%) received trastuzumab as monotherapy. Most of the patients received a taxane in the 1st line setting in addition to trastuzumab (137 patients, 72%), while 27 patients (14%) received anthracyclines.

Median follow-up for all patients was 66 months, while for patients treated with trastuzumab in the 1st line median follow-up was 51 months. Totally, 137 patients died among all patients, while 151 of the 191 patients treated with trastuzumab in the 1st line demonstrated tumor progression. Median survival was significantly longer in HER2-positive patients treated with trastuzumab (median survival 50.4 months, 95% Confidence Interval [CI]: 39.4-61.4) compared to HER2-negative patients (median survival 35.3 months, 95% CI: 30.9-39.6, log-rank, p=0.007). TTP was 14 months (95% CI: 9.6-18.5) for HER2-positive patients treated with 1st line trastuzumab, as compared to 10.3 months (95% CI: 5.6-15.0) for HER2-negative patients. This difference was not statistically significant (log-rank, p=0.24), probably due to the small number of patients. ER, PgR, Ki67, PTEN and *PIK3CA* data were presented in detail in a previous publication [22].

### Associations between examined markers

**Figure 2:**
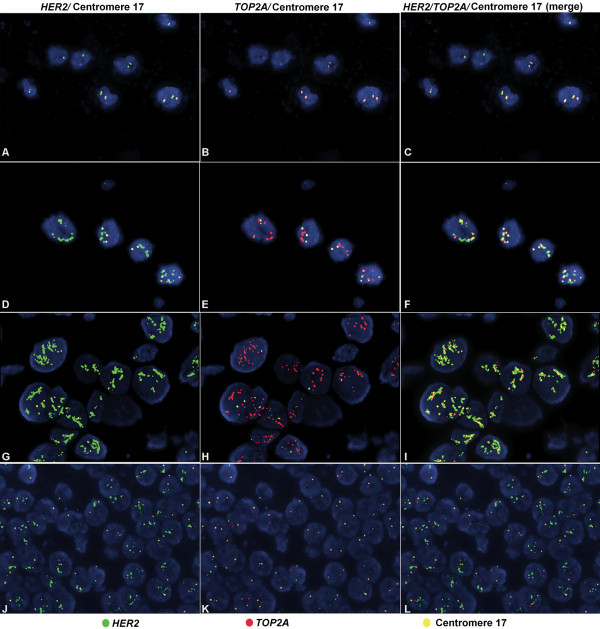
**Fluorescence in situ hybridization (FISH) in breast cancer cell lines and invasive breast carcinomas (IBC).** FISH in breast cancer cell lines (**A**-**F**) and IBC cases (**G**-**L**) for the *HER2* and *TOP2A* genes using the *HER2*/*TOP2A*/CEN17 triple color probe. The MDA-MB-231 cell line showed normal status of the *HER2* and *TOP2A* genes (**A**-**C**), whereas in the SK-BR-3 cell line, co-amplification of the *HER2* and *TOP2A* genes was found (**D**-**F**). IBC case showing simultaneous amplification of the *HER2* and *TOP2A* genes (**G**-**I**); IBC case with amplification of the *HER2* gene, deletion of the *TOP2A* gene and normal chromosome 17 status (**J**-**L**). Panels **A**, **D**, **G** and **J** show *HER2* and centromere 17 hybridization signals; Panels **B**, **E**, **H** and **K** show *TOP2A* and centromere 17 hybridization signals, where as panels **C**, **F**, **I** and L depict merged *HER2*, *TOP2A* and centromere 17 signals. Magnification x1000.

*TOP2A* gene alterations were assessed in 221 tumors, 25% of which were amplified and 12% were deleted. Tumors that were not *TOP2A* amplified or deleted were analyzed as a separate group (*TOP2A* non-amplified tumors). As expected, *TOP2A* gene amplification and HER2-negative status were mutually exclusive. Representative FISH images of the evaluated cell lines and invasive breast carcinoma cases are presented in Figure 2. The majority of *TOP2A* gene deletions were seen in the HER2-positive group (21 of the 26 cases), while 43% of HER2-positive tumors were not amplified for *TOP2A* (Table 2). A significant association of *TOP2A* gene status with HER2 status was found (Fisher’s exact test, p<0.001). Moreover, *TOP2A* gene amplification was more frequent in ER-negative tumors (28% in ER-negative vs. 24% in ER-positive, p=0.017), whereas no such association was found with Ki67 (p=0.47) or PTEN protein expression (p=0.12). *TOP2A* gene amplification was negatively associated with the presence of *PIK3CA* mutations (28% in WT vs. 14% in mutated, p=0.035), the distribution however, of *PIK3CA* mutations in the HER2-positive group did not differ between *TOP2A* amplified and non-amplified tumors (12% vs. 23%, p=0.25).

**Table 2:** **Association of*TOP2A*and TopoIIa with HER2 status**

		**HER2 status**				**Fisher’s exact p**
		**Positive**		**Negative**		
		**N**	**%**	**N**	**%**	
*TOP2A* (FISH) n=221	Amplified	55	41.0	0	0	<0.001
	Deleted	21	15.7	5	5.7	
	Non-amplified	58	43.3	82	94.3	
TopoIIa (IHC) n=193	Negative	39	33.9	29	37.2	0.65
	Positive	76	66.1	49	62.8	

Concerning TopoIIa protein expression, the majority of cases were positive (125 of 193 cases assessed, 65%), but no association with HER2 status was found (p=0.65). TopoIIa protein positivity was associated with ER-positive status (72% in ER-positive vs. 49% in ER-negative, p=0.005), high Ki67 expression (68% in high vs. 32% in low, p=0.004) and positive PTEN protein status (80% in positive vs. 58% in negative, p=0.003). The majority of the *PIK3CA* mutations in this cohort (28 of 34) were encountered in tumors with positive TopoIIa protein expression (p=0.015).

For 189 tumors, both *TOP2A* gene status and TopoIIa protein expression were assessed (Figure 1), but no significant association was found between the two (p=0.11). More specifically, 37 among 49 *TOP2A* amplified tumors were TopoIIa-positive (76%), while 73 among 117 *TOP2A* non-amplified tumors were TopoIIa-positive (62%). *TOP2A* gene deletions were equally distributed according to TopoIIa protein expression (11 TopoIIa-negative cases vs. 12 TopoIIa-positive cases).

### Effects of TOP2A gene alterations and TopoIIa protein expression on the outcome of MBC patients treated with trastuzumab

Survival analysis was performed in the 1st line treated subpopulation (191 of the 225 patients treated with trastuzumab), as defined in the statistical analysis section. Since *TOP2A* gene amplification and HER2-negativity were found to be mutually exclusive, we examined in univariate analysis the association of *TOP2A* gene status with outcome in the HER2-positive subgroup only. *TOP2A* gene status was not associated with TTP (Wald’s p=0.14), however, *TOP2A* non-amplified and deleted tumors were associated with increased risk for death (Hazard Ratio [HR]=2.16, 95% CI: 1.20-3.88, Wald’s p=0.010 and HR=2.67, 95% CI: 1.27-5.62, p=0.009, respectively) compared to amplified tumors (Table 3).

**Table 3:** Univariate Cox regression models for TOP2A expression according to HER2 status

	**HER2-positive**				**HER2-negative**			
	**Events**	**HR**	**95% CI**	**Wald’s p**	**Events**	**HR**	**95% CI**	**Wald’s p**
**TTP**								
*TOP2A* (FISH)								
Deleted vs. Amplified	16 vs. 36	1.58	0.87-2.87	0.13				
Non-amplified vs. Amplified	43 vs. 36	1.51	0.96-2.37	0.07				
TopoIIa (IHC)								
Positive vs. Negative	57 vs. 27	0.94	0.60-1.49	0.80	31 vs. 17	1.01	0.55-1.83	0.98
								
**Survival**								
*TOP2A* (FISH)								
Deleted vs. Amplified	12 vs. 17	2.67	1.27-5.62	0.009				
Non-amplified vs. Amplified	33 vs. 17	2.16	1.20-3.88	0.010				
TopoIIa (IHC)								
Positive vs. Negative	38 vs. 19	0.71	0.41-1.24	0.22	23 vs. 14	0.70	0.36-1.37	0.30

*CI* confidence interval, *HR* hazard ratio, *TTP* time to progression.

Empty cells: Non-applicable.

TopoIIa protein expression was not associated with either TTP (Wald’s p=0.80 for HER2-positive and p=0.98 for HER2-negative patients) or survival (Wald’s p=0.22 for HER2-positive and p=0.30 for HER2-negative patients). Tests for interaction of TopoIIa protein expression with HER2 status were not significant (Wald’s p>0.05, for both TTP and survival). Interaction tests of *TOP2A* gene status with HER2 status were not applicable, since *TOP2A* gene amplification and HER2-negativity were found to be mutually exclusive.

Since, subgroups defined by ER/PgR status are of clinical interest, we also examined whether there was a significant interaction between ER/PgR status and *TOP2A* gene status or TopoIIa protein expression. No significant interactions were found (Wald’s p values >0.05).

### Interaction of TOP2A gene alterations with anthracycline treatment

In the multivariate setting, we examined the predictive role of *TOP2A* gene expression to anthracycline treatment administered either in the adjuvant or the 1st line metastatic setting. Ninety-three patients received anthracyclines as adjuvant and/or 1st line treatment. The majority of them (64 cases, 69%) were HER2-positive. Concerning *TOP2A* gene expression in the anthracycline-treated/HER2-positive subgroup, 25 patients were amplified, 29 were non-amplified and 10 cases had a *TOP2A* deletion.

**Figure 3:**
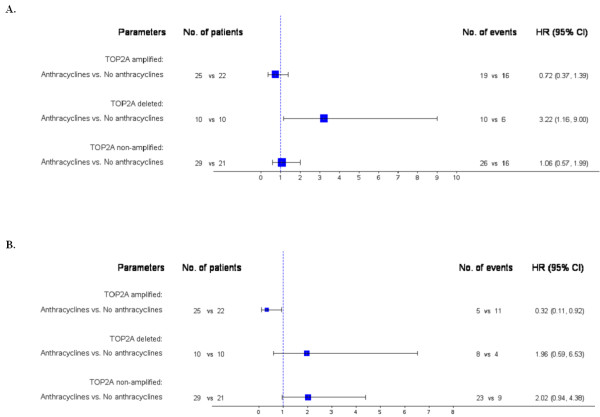
**Forest plots from multivariate Cox regression models in the HER2-positive patients.A**: Time to progression (N=117). Among the *TOP2A* deleted subgroup, anthracycline-treated patients were associated with increased risk for progression. **B**: Survival (N=117). Among the *TOP2A* amplified subgroup, anthracycline-treated patients were associated with decreased risk for death.

In the HER2-positive subgroup, a significant interaction of *TOP2A* with anthracycline treatment (either in the adjuvant or the 1st line setting) was observed both for TTP (Wald’s p=0.055) and survival (Wald’s p=0.015), while no clinicopathological parameters were retained in the final model (Figure 3). In terms of TTP (Figure 3A), among the *TOP2A* deleted subgroup, anthracycline-treated patients were associated with increased risk for progression, while in terms of survival (Figure 3B), among the *TOP2A* amplified subgroup, anthracycline-treated patients were associated with decreased risk for death. No significant interactions were found between TopoIIa protein expression and anthracycline treatment (Wald’s p values >0.05).

**Figure 4:**
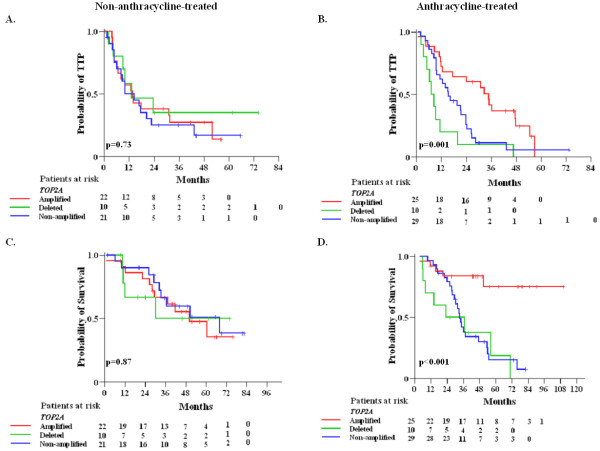
**Kaplan-Meier curves according *TOP2A *gene status and anthracycline treatment.** Time to progression (TTP, **A**-**B**) and survival (**C**-**D**) according to *TOP2A* gene status in the HER2-positive patients stratified by anthracycline treatment.

In parallel, for patients not treated with anthracyclines there were no significant differences in TTP between deleted and non-amplified versus amplified tumors (HR=0.76, 95% CI: 0.29-1.96, Wald’s p=0.57 and HR=1.23, 95% CI: 0.61-2.47, p=0.56, respectively) (Figure 4A). For anthracycline-treated patients, deleted and non-amplified tumors were associated with increased risk for progression compared to amplified tumors (HR=3.42, 95% CI: 1.57-7.46, Wald’s p=0.002 and HR=1.83, 95% CI: 1.00-3.34, p=0.050, respectively) (Figure 4B). Similarly, in terms of survival the same associations were observed. Among anthracycline-treated patients, *TOP2A* deleted and non-amplified tumors had increased risk for death compared to amplified tumors (HR=6.94, 95% CI: 2.26-21.34, Wald’s p=0.001 and HR=5.33, 95% CI: 2.02-14.12, p=0.001, respectively) (Figure 4D). In patients not treated with anthracyclines no such differences in survival were observed (Figure 4C). In the contrary, no significant differences in TTP and survival were observed between anthracycline- and non-anthracycline-treated patients in the HER2-positive subgroup when *TOP2A* gene status was not taken into account (log-rank, p=0.67 for TTP and p=0.57 for survival).

Repeating the analysis in HER2-positive patients that had received anthracyclines in the adjuvant setting (52 of the 64 anthracycline-treated/HER2-positive patients) the results were identical to the ones presented in Figure 3. Among the *TOP2A* deleted subgroup, adjuvant anthracycline-treated patients were associated with increased risk for progression (HR=3.25, 95% CI: 1.16-9.09, Wald’s p=0.025), while among the *TOP2A* amplified subgroup, adjuvant anthracycline-treated patients were associated with decreased risk for death (HR=0.27, 95% CI: 0.08-0.94, Wald’s p=0.041).

In multivariate analysis of the total cohort, the significance of *TOP2A* in anthracycline-treated patients remained, resulting in similar associations for both TTP and survival (Additional file 1 and Additional file 2) to the ones seen in the HER2-positive subgroup.

Finally, when examining in the models any *TOP2A* alteration (deletions or amplifications) versus non-amplification, the interaction with anthracycline treatment was significant only in the case of the HER2-positive subgroup in terms of survival (p=0.042), while among the anthracycline-treated patients, tumors with *TOP2A* alterations were associated with improved survival compared to the non-amplified tumors.

## Discussion

To our knowledge, the present study is one of the first to evaluate the role of *TOP2A* gene amplification and TopoIIa protein expression in the outcome of patients treated with trastuzumab-based regimens for MBC. The most important evidence provided herein is that *TOP2A* gene amplification is a favorable prognostic factor in HER2-positive patients treated with trastuzumab. Patients with HER2-positive/*TOP2A* non-amplified or deleted tumors did not seem to benefit from trastuzumab-based regimens and had an unfavorable outcome compared to *TOP2A* amplified tumors, in line with recent reports on *TOP2A* gene dosage [35] and *TOP2A* gene amplification [36].

The role of the *TOP2A* gene has mainly been examined in relation to anthracycline treatment. Co-amplification of *HER2* and *TOP2A* was associated with favorable response to anthracycline-based therapy of locally advanced breast cancer [12]. The results of our study did not appear, at first, to be associated with the administration of anthracyclines, since only 12% of our patients had received such treatment in the 1st line metastatic setting. However, 39% of all patients and 43% of the HER2-positive ones had received anthracyclines in the adjuvant setting. Upon further analysis of our data, taking into account adjuvant and/or 1st line anthracycline treatment, a significant interaction of *TOP2A* with anthracycline treatment was observed both for TTP and survival. In terms of survival among the *TOP2A* amplified subgroup, anthracycline-treated patients were associated with decreased risk for death. It appears therefore that the improvement in survival of the *TOP2A* amplified subgroup treated with trastuzumab is probably due to the concurrent or previous exposure of the patients to anthracyclines, rather than the effect of the trastuzumab treatment itself. Furthermore, when *TOP2A* amplified patients treated with anthracyclines in the adjuvant setting were analyzed separately, they were found to have decreased risk for death, suggesting that even history of adjuvant anthracycline treatment results in survival advantage of *TOP2A* amplified patients treated with trastuzumab.

There is a very recent report from the Breast Cancer International Research Group (BCIRG) 006 trial [37] and an additional retrospective analysis of almost 5,000 patients [13] regarding the efficacy of trastuzumab in breast cancer patients with *HER2* and *TOP2A* co-amplification. The first study is one of the largest randomized trials, which confirmed the role of trastuzumab in the adjuvant setting. The most interesting aspect of the BCIRG 006 trial is that it included a non-anthracycline regimen (docetaxel, carboplatin and trastuzumab), which was compared to AC-T (doxorubicin, cyclophosphamide, followed by docetaxel) with or without trastuzumab. This study demonstrated that the DFS benefit conferred by AC-T without trastuzumab in HER2-positive breast cancer patients is actually restricted to *TOP2A* co-amplified malignancies, which constituted a subset (35%) of the HER2-positive cancers, and is virtually indistinguishable from the benefit achieved by the addition of trastuzumab. Importantly, this same benefit (found in the *TOP2A* co-amplified subset) could also be attained by a non-anthracycline regimen in combination with trastuzumab, thus avoiding the toxicities seen with anthracyclines. In our study, trastuzumab was given to advanced-stage HER2-positive breast cancer patients in the metastatic setting, our findings should not therefore be compared to those of the BCIRG 006 trial [37].

Recent studies support the role of TopoIIa protein expression, rather than *TOP2A* gene amplification, as a predictor of response to anthracycline-based chemotherapy in the adjuvant setting [16]. It is of note, that TopoIIa protein overexpression has been reported in HER2-positive, as well as HER2-negative tumors, independently of *TOP2A* gene amplification [38]. The latter finding has also been shown in our study; TopoIIa protein overexpression however, was not associated with either TTP or survival.

TopoIIa protein overexpression was however associated with ER-positive status and high Ki67 expression, partly in line with previous reports, since TopoIIa protein expression had been shown to be associated with ER-positive status [39] and the Ki67 proliferation index [40]. To the best of our knowledge, the associations between TopoIIa and PTEN protein expression, as well as *PIK3CA* mutation presence are new findings in breast cancer tissue series, meriting further investigation for their biological importance. Of note, TopoIIa protein is upregulated in proliferating normal and cancer cells, in order to participate in the cell duplication process [41]. Hence, with the widely used cut-off of 5% positive neoplastic cells to assess TopoIIa protein positivity, tumors are found to be positive for TopoIIa in the absence of underlying amplification of the corresponding gene.

Alterations of the *TOP2A* gene mostly happen in HER2-positive tumors, however *TOP2A* does not always follow the amplification fate or rate of the *HER2* amplicon, since it is not always included in the so called “smallest region of amplification” next to *HER2*[36,42], while it may also be deleted in the presence of *HER2* amplification, as observed here and elsewhere [10]. Thus, at least in a subset of *TOP2A* amplified tumors, the mechanism driving *TOP2A* amplification may be different than the one resulting in *HER2* amplification [9,10,43], as shown by the far lower ratio of *TOP2A* signals in comparison to *HER2* signals [10]. In addition, *TOP2A* may also be amplified or deleted in the absence of *HER2* amplification, further supporting the view of distinct and possibly multiple mechanisms, resulting in alterations of this gene. The absence of *TOP2A* amplification and the presence of deletions may practically have the same unfavorable impact on the outcome of HER2-positive patients, as shown in this study. With respect to gene deletions, it should be noted that the way markers are scored with FISH on FFPE sections it is unavoidable to obtain false positive results (deletions), due to nuclear truncations that interfere with the number of fluorescent signals to be counted per nucleus in a mostly unpredictable manner. Hence, although TOP2A gene deletions may indeed occur, the results concerning this FFPE-FISH marker, in the present and in the previously published studies, should be interpreted with caution.

In most of the published series, *TOP2A* gene amplification or deletion was a rare event in HER2-negative patients [44]. Only in four studies [10,16,18,45], the rate of *TOP2A* alterations was considerably greater than the 1% to 2% range reported in all other studies. In line with the majority of the published data we did not find HER2-negative patients with *TOP2A* gene amplification.

## Conclusions

In conclusion our study is one of the first to examine the role of the *TOP2A* gene in the field of trastuzumab-based treatment in MBC. We have evaluated the role of *TOP2A* gene amplification and TopoIIa protein expression and we have shown that *TOP2A* gene amplification is a favorable prognostic factor in HER2-positive MBC patients treated with trastuzumab, such an effect however, appears to rather be related to treatment with anthracyclines. In advanced-stage HER2-positive breast cancer patients treated with trastuzumab, *TOP2A* amplification appears to be a strong predictive factor for improved survival in patients with concurrent or previous exposure to anthracyclines. Nevertheless, given the small size and the retrospective nature of our study, these data have to be viewed as hypothesis generating and need to be further explored and validated in larger cohorts of patients treated in the context of randomized trials. We are currently investigating these associations in patients included in a large adjuvant phase III trial conducted by our Group.

## Abbreviations

CEN17: Centromere 17; CI: Confidence interval; ER: Estrogen receptor; FFPE: Formalin-fixed paraffin-embedded; HER2: Human epidermal growth factor receptor 2; HR: Hazard ratio; IHC: Immunohistochemistry; Ki67: Antigen Ki67; MBC: Metastatic breast cancer; OS: Overall survival; PIK3CA: Phosphoinositide-3-kinase, catalytic, alpha polypeptide; PFS: Progression-free survival; PgR: Progesterone receptor; PTEN: Phosphatase and tensin homolog deleted on chromosome 10; SNP: Single nucleotide polymorphism; TOP2A: Topoisomerase II alpha (gene amplification); TopoIIa: Topoisomerase II alpha (protein expression); TTP: Time to progression; TMA: Tissue microarray.

## Competing interests

The authors declare that they have no competing interests.

## Authors’ contributions

GF conceived of the study, participated in its design and coordination, participated in the clinical management of the patients, contributed to the collection of the tumor tissue samples analyzed in the study and drafted the manuscript. CC conceived of the study, participated in its design and coordination, participated in the clinical management of the patients, contributed to the collection of the tumor tissue samples analyzed in the study and drafted the manuscript. MB carried out the TMA construction and the IHC and FISH assays and helped to draft the manuscript. VK carried out the molecular studies and helped to draft the manuscript. AGE performed the statistical analysis and helped to draft the manuscript. IX participated in the clinical management of the patients and contributed to the collection of the tumor tissue samples analyzed in the study. AB carried out the immunoassays. GP participated in the clinical management of the patients and contributed to the collection of the tumor tissue samples analyzed in the study. NX participated in the clinical management of the patients and contributed to the collection of the tumor tissue samples analyzed in the study. IP carried out the immunoassays. AK participated in the clinical management of the patients and contributed to the collection of the tumor tissue samples analyzed in the study. PP participated in the clinical management of the patients and contributed to the collection of the tumor tissue samples analyzed in the study. DB participated in the clinical management of the patients and contributed to the collection of the tumor tissue samples analyzed in the study. DVS participated in the clinical management of the patients and contributed to the collection of the tumor tissue samples analyzed in the study. KTK conceived of the study, participated in its design and coordination and drafted the manuscript. All authors read and approved the final manuscript.

## Supplementary Material

Additional file 1 Forest plots from multivariate Cox regression models in the total study population: time to progression (A, n=181) and survival (B, n=172). Click here for file

Additional file 2 **Kaplan-Meier curves for time to progression (TTP, A-B) and survival (C-D) according to*TOP2A *gene status in the total study population stratified by anthracycline treatment.** Click here for file
