# Supplementary figures and images for: Topoisomerase II alpha gene amplification is a favorable prognostic factor in patients with HER2-positive metastatic breast cancer treated with trastuzumab
Source: J Transl Med. 2012 Oct 23;10:212. doi: 10.1186/1479-5876-10-212 (PMC3499161; doi:10.1186/1479-5876-10-212)

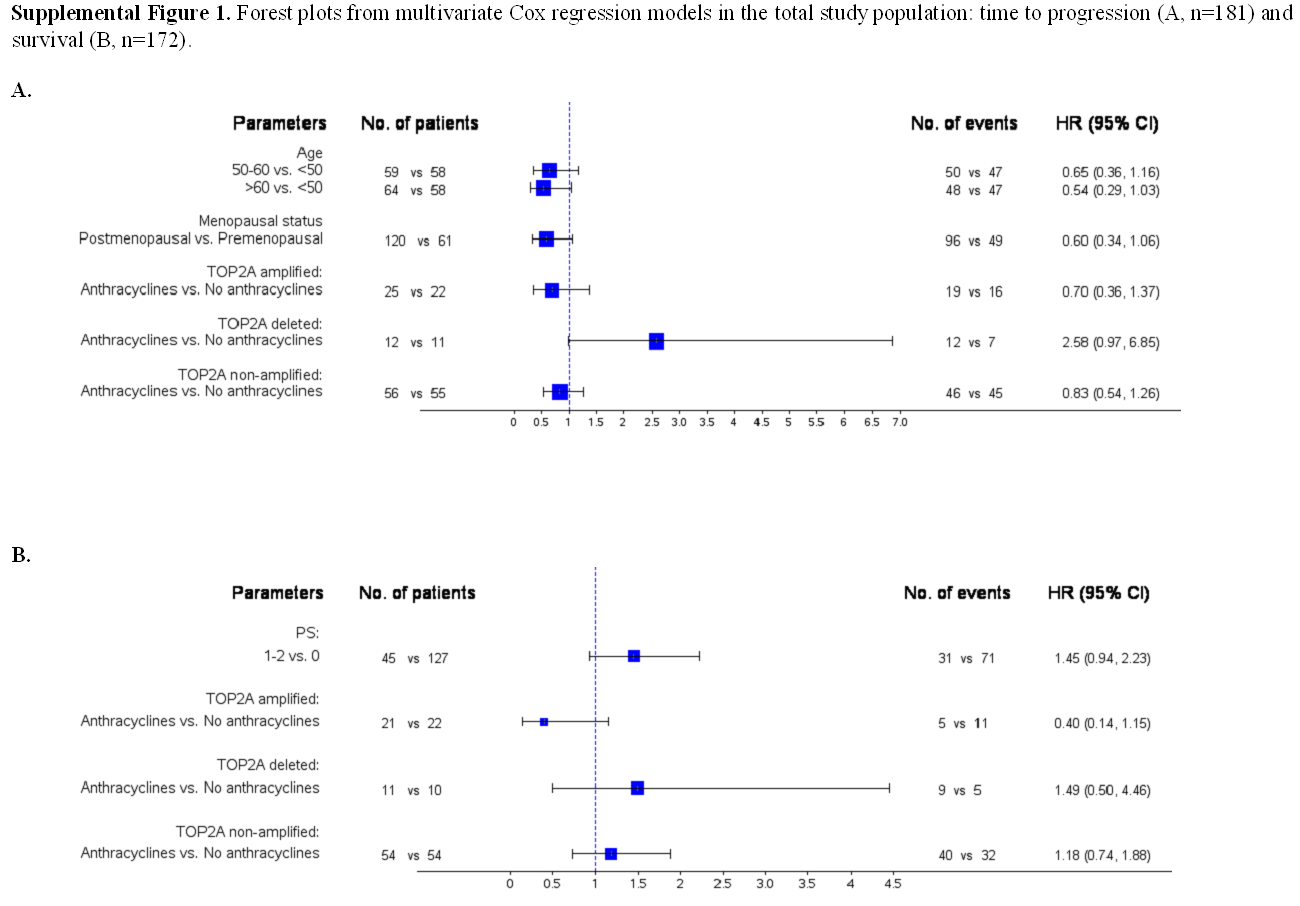

Supplement: Additional file 1 — Forest plots from multivariate Cox regression models in the total study population: time to progression (A, n=181) and survival (B, n=172). [file 1479-5876-10-212-S1.tiff]

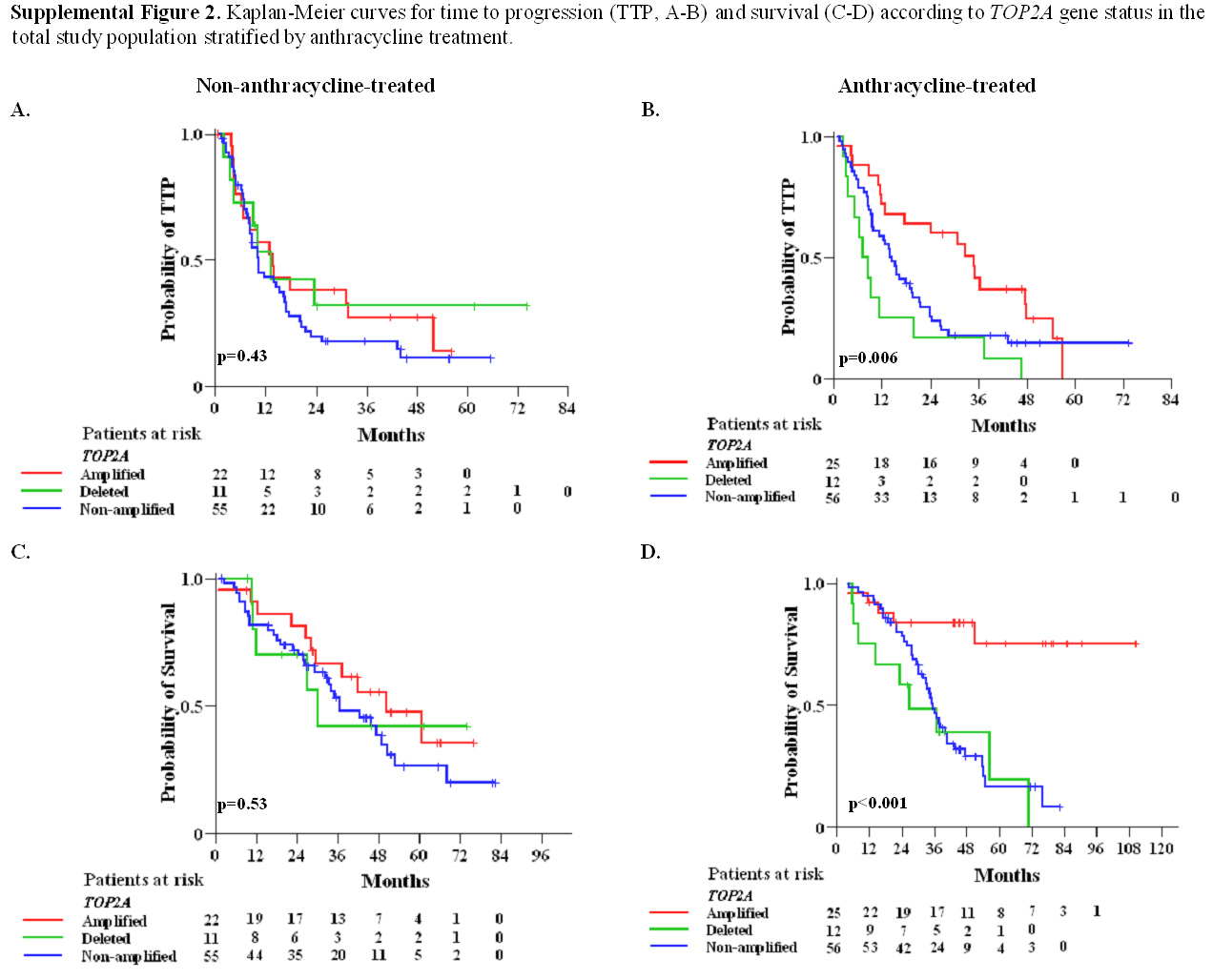

Supplement: Additional file 2 — Kaplan-Meier curves for time to progression (TTP, A-B) and survival (C-D) according toTOP2A gene status in the total study population stratified by anthracycline treatment. [file 1479-5876-10-212-S2.tiff]
